# Directed conjunctival biopsy and impact of histologic sectioning methodology on the diagnosis of ocular sarcoidosis

**DOI:** 10.1186/1869-5760-4-8

**Published:** 2014-03-18

**Authors:** Kelly M Bui, Jose M Garcia-Gonzalez, Sarju S Patel, Amy Y Lin, Deepak P Edward, Debra A Goldstein

**Affiliations:** 1 University of Illinois Eye and Ear Infirmary, Chicago, IL 60612, USA; 2 Wilmer Eye Institute, Johns Hopkins University, Baltimore, MD 21287, USA; 3 King Khaled Eye Specialist Hospital, Riyadh 11642, Kingdom of Saudi Arabia; 4 Northwestern University Feinberg School of Medicine, Chicago, IL 60611, USA

**Keywords:** Biopsy, Conjunctiva, Sarcoidosis, Uveitis, Histology, Pathology

## Abstract

**Background:**

Sarcoidosis is an idiopathic, multi-system, granulomatous disease with well-described ocular manifestations. However, other uveitic etiologies can manifest in a similar fashion, and ocular disease may precede systemic manifestations. Definitive diagnosis requires histologic confirmation of non-caseating granulomatous inflammation. This study reports the diagnostic yield of directed biopsy of conjunctival follicles in patients with uveitis suspected to be secondary to sarcoidosis, and compares an institutional standard tissue sectioning method to a multi-plane technique.

**Results:**

A retrospective analysis was performed of all patients who underwent directed conjunctival biopsy for suspected ocular sarcoidosis. A total of eight patients were identified; all were females. Directed conjunctival biopsy was positive in three of seven patients using standard histologic processing method, a yield of 43%. Using the multi-plane technique increased the cumulative yield to 63%.

**Conclusions:**

Directed conjunctival biopsy is a minimally invasive, cost-effective, and moderately high yield method of diagnosing ocular sarcoidosis. Using a multi-plane sectioning method may increase biopsy yield when standard sectioning techniques are negative.

## Background

Sarcoidosis is an idiopathic, multi-system, granulomatous disease, affecting people of all ethnicities, gender, and age groups. Definitive diagnosis requires histologic evidence of non-caseating, granulomatous inflammation, commonly from biopsy of mediastinal lymph nodes. Another potential biopsy site is the conjunctiva, with a reported diagnostic yield ranging from 20% to 70% with blind biopsies [1-4] and 36% to 75% with directed biopsies [5-8]. This wide range may be explained by different tissue sectioning and analysis techniques; however, few studies describe their histologic methodology.

This study highlights the diagnostic utility of directed conjunctival biopsy and compares an institutional standard tissue sectioning method to a multi-plane sectioning technique.

## Methods

This study is a retrospective case series of patients who underwent directed conjunctival biopsy for suspected ocular sarcoidosis at the University of Illinois, Chicago Uveitis clinic between January 1999 and August 2011. This study was approved by the University of Illinois institutional review board. Directed biopsies were performed on conjunctival follicles suspected to represent sarcoid granulomas. Patients were excluded from the study if the biopsy was performed blindly or in the absence of visible conjunctival follicles.

### Biopsy technique

**Figure 1:**
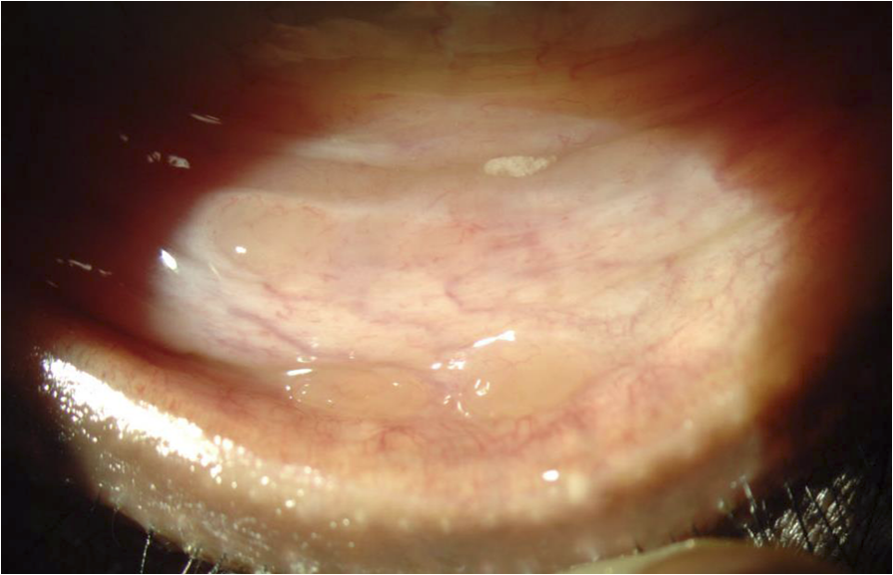
Conjunctival follicles suggestive of granulomas.

Topical proparacaine hydrochloride 0.5% was instilled in the inferior cul-de-sac of the involved eye. Conjunctival follicle(s) was identified and marked (Figure 1). Using a 25-gauge needle on a syringe, 1% lidocaine was injected into the subconjunctival space, ballooning the previously marked area. The lower eyelid was then retracted and the inferior fornix was grasped away from the identified follicle(s) with 0.12 forceps. The strip of involved conjunctiva was excised using Westcott scissors, flattened and placed onto a piece of filter paper to prevent scrolling of the specimen. Following a few seconds of air-drying, the paper was placed into 10% neutral buffered formalin. All biopsies were performed or supervised by the same surgeon (DAG).

### Histologic methods

**Figure 2:**
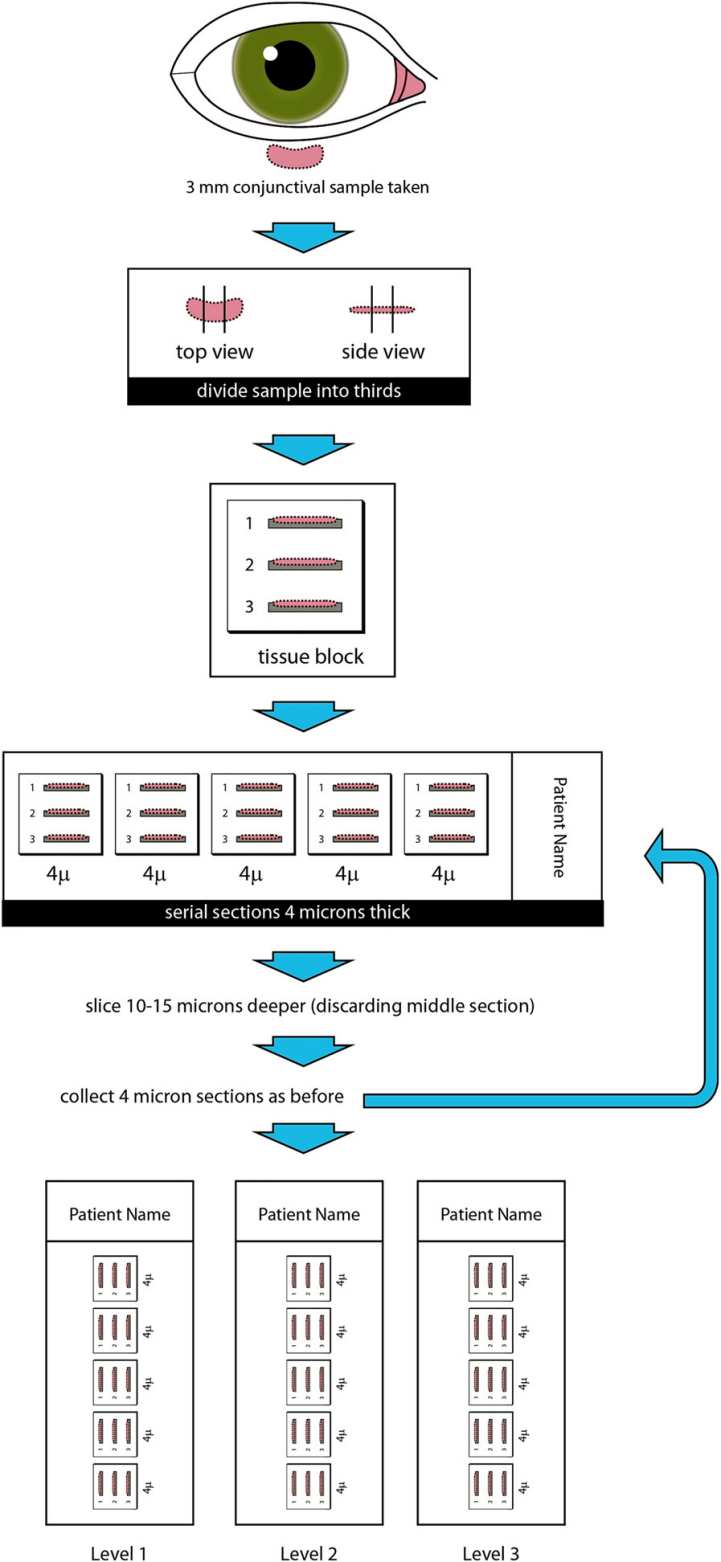
Multi-plane sectioning technique.

Tissue samples were embedded in paraffin, sectioned, and stained with hematoxylin and eosin. The biopsy size was determined based on that reported during gross examination in the pathology report. Two different sectioning techniques were used. For the standard technique, a ribbon of tissue with five sections was cut from a block at one level and placed on a glass slide. For the multi-plane technique, the standard sectioning process was repeated at 3 different levels 15 μm apart (Figure 2). The standard sectioning gave an average of 5 sections to analyze while the multi-plane method gave an average of 15 sections per sample.

**Figure 3:**
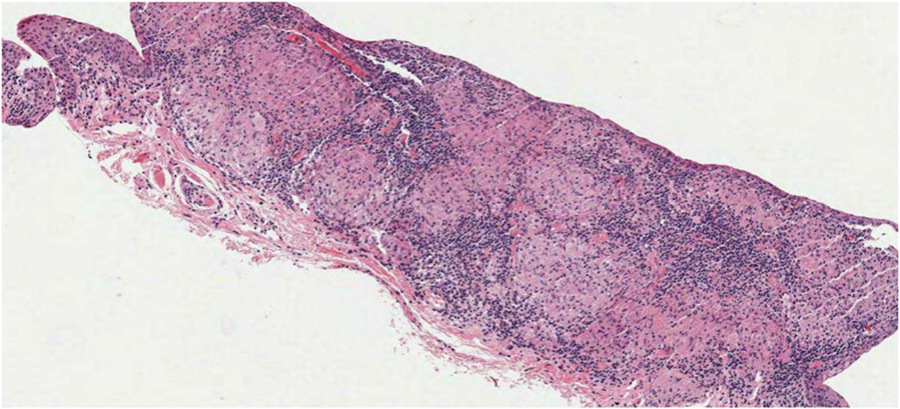
Hematoxylin-eosin slide demonstrating discrete non-caseating granulomas.

A biopsy was defined as positive if discrete non-caseating granulomas were identified in at least one section (Figure 3). If the initial biopsy result using the standard technique was negative, the tissue block underwent repeat sectioning using the multi-plane technique.

All specimens were stained with Ziehl-Nielson and Gomori methenamine silver stains to rule out infectious granulomas caused by acid-fast organisms and fungi, respectively. Interpretation of histologic data was performed by one of two ocular pathologists.

### Data analysis

Descriptive data are presented as means and percentages. Given the small sample size, a non-normal distribution was assumed. Means were compared using the Mann-Whitney *U* test, while categorical data was compared using chi-squared analysis with Fischer’s exact *P* values calculated. Epi Info™ 3.5.3 (Center for Disease Control, Atlanta, GA, USA) was used in the data analysis.

## Results

A total of eight patients with suspected sarcoidosis underwent directed conjunctival biopsy. All patients were female, with a mean age of 43.5 years (range 20 to 62 years) at onset of ocular symptoms. Seven patients (87.5%) were African-American and one was Asian. Five patients (62.5%) had chronic granulomatous anterior uveitis, and three (37.5%) had granulomatous panuveitis (Table 1).

**Table 1:** Clinical, laboratory, radiologic, histologic data

**Patient no.**	**Age**	**Sex**	**Clinical features**	**ACE**	**Lysozyme**	**CXR**	**Chest CT**	**Treatment prior to biopsy**	**Standard sectioning**	**Multi-plane sectioning**
1	31	F	CGIC	Normal	Elevated	POS	ND	None	ND	POS
2	52	F	Panuveitis (CGIC, MFC, Retinal granulomas + Periphlebitis)	Elevated	Elevated	POS	POS	None	NEG	POS
3	62	F	Panuveitis (Vitreous, retinal, and choroidal granulomas)	ND	ND	POS	ND	None	POS	ND
4	40	F	CGIC (Retinal granulomas)	Normal	Elevated	POS	POS	None	POS	ND
5	62	F	CGIC	Normal	Normal	NEG	POS	MTX, prednisone	POS	ND
6	51	F	CGIC	Normal	Normal	NEG	ND	None	NEG	NEG
7	20	F	CGIC, MFC	Normal	Normal	NEG	ND	Prednisolone acetate	NEG	NEG
8	30	F	CGIC	Elevated	Elevated	NEG	ND	Prednisolone acetate	NEG	NEG

CGIC, chronic granulomatous iridocyclitis; MFC, multifocal choroiditis; ACE, angiotensin converting enzyme; CXR, chest X-ray; CT, computed tomography; ND, no data; POS, positive; NEG, negative; MTX, methotrexate.

Eight unilateral directed biopsies were performed, all from the inferior fornix. Biopsy samples ranged in size from 4 × 2 × 1 mm to 8 × 1.5 × 1 mm (mean 5 × 3 × 1 mm). There was no statistically significant difference in size of tissue samples between positive and negative biopsies (*P* = 0.68).

Seven of eight conjunctival samples initially underwent standard sectioning. A total of five samples underwent multi-plane sectioning, one as an initial method and four as repeat sectioning after a negative biopsy result.

Directed conjunctival biopsy was positive in three of seven patients using standard histologic processing method, a yield of 43% (Table 1). One of eight biopsies was initially analyzed using the multi-plane sectioning technique and was positive. Of the four biopsies that were negative via the standard histologic method, one additional positive case was identified when re-sectioned using the multi-plane method. When both sectioning techniques were taken into consideration, there was a cumulative yield of 63%. None of the five patients with biopsies consistent with sarcoidosis were on topical corticosteroids at the time of biopsy, while two patients with negative biopsies were on topical corticosteroid drops for at least 1 month before biopsy. Only one of eight patients was on systemic therapy (methotrexate for 3 weeks and 60 mg of prednisone for 1 week) before conjunctival biopsy, and biopsy was positive.

None of the eight patients had an established diagnosis of sarcoidosis prior to ocular symptoms. Two of five patients with positive biopsy had dermatologic manifestations consistent with cutaneous sarcoidosis (indurated plaques on arms and legs in one patient and thickening and edema of the eyelid in another patient); both had subsequent positive skin biopsy for sarcoidosis. There was no other organ involvement in the three negative biopsy patients.

Four of five patients with a positive biopsy had chest X-rays (CXR) consistent with sarcoidosis (Table 1), including calcified nodules suggestive of old granulomatous disease or prominent hilar adenopathy, while none of the three patients with a negative biopsy had findings on CXR to suggest sarcoidosis (*P* = 0.07). Chest computed tomography (CT) was obtained in three patients who had a positive conjunctival biopsy. All three had chest CT findings consistent with sarcoidosis, including one patient with an initial unremarkable CXR. Considering all chest imaging modalities, all five patients with positive conjunctival biopsy had imaging consistent with sarcoidosis; which was not seen in any of the three patients with negative biopsies (*P* = 0.02).

Of the positive biopsy patients, only four had available results of angiotensin-converting enzyme (ACE) and lysozyme testing. (Table 1). Of these four patients, three had elevated lysozyme levels and one had elevated ACE levels. One of three patients with normal ACE levels was on an ACE inhibitor, although lysozyme level was also normal. Of the biopsy negative patients, only one had elevated ACE and lysozyme levels.

## Discussion

Uveitis may be the presenting sign of sarcoidosis, and is present in about 20% of cases [9]. Although typical ocular signs such as granulomatous iridocyclitis, retinal periphlebitis, and chorioretinitis are clinical clues to the diagnosis of sarcoidosis, other diagnoses such as tuberculosis, syphilis, and primary intraocular lymphoma may present in a similar fashion [10-12]. Conjunctival follicles have been reported in 7% to 17% of patients with ocular sarcoidosis [13,14]. Crick et al. first popularized directed biopsy of these follicles, with a yield of 24% to 36% [5,15]. Since then, debate has remained about performing directed versus non-directed biopsies, and little consensus exists regarding the optimal method of histologic sectioning. In the literature, some institutions performed 5 to 6 serial sections at three levels [1] whereas others performed 10 serial sections at 6 different levels for a total of 60 sections [2]. No studies to date have evaluated the effect of number of serial sections on conjunctival biopsy yield, although the importance of careful sectioning has been previously emphasized [1,5,15,16].

Merritt et al. [7] reported a 75% yield when they performed directed conjunctival biopsy in 16 patients with suspected sarcoidosis. All 16 biopsies received a minimum of 10 serial sections and in 3 biopsy samples, granulomas were only detected in deeper sections. Takayama et al. [17] found a similar result in transbronchial biopsy where step sectioning of specimens increased the yield from 38% to 47% in patients with stage I lung disease and from 57% to 82% in stage II disease.

The recovery of granulomas with conjunctival biopsy using careful sectioning technique approaches the yield obtained from other tissue sites [16,17], especially when conjunctival pathology such as nodules, follicles, or fibrous scarring is present [4,12,18]. No false positives have been reported in previous studies [18,19]. Spaide and Ward [20] found histologic evidence of sarcoidosis in 34 of 47 patients; 40% (19 of 47) were positive on conjunctival biopsy and 66% (31 of 47) on transbronchial biopsy. Leavitt and Campbell [16] reported the utility and cost-effectiveness of conjunctival biopsy compared to other biopsy sites. They found a 51% yield with conjunctival biopsy. Twenty patients with negative biopsy underwent an additional 28 biopsies from other tissue sites; 71% were positive. Twenty-one patients with positive conjunctival biopsy had 19 biopsies from other tissue sites, some predating the conjunctival biopsy; 90% were positive. He concluded that patients with positive conjunctival biopsy could have been spared a total of 17 procedures with an estimated saving of $81,350 to $1,121,150 in 1997, if diagnosis had relied only on conjunctival biopsy. The cost of a conjunctival biopsy at the University of Illinois in 2012 was $843 ($315 for biopsy, $528 for pathology) compared to $1,774 ($898 biopsy; $876 pathology) for a transbronchial biopsy and $5,447 ($4,017 biopsy; $1,430 pathology) for a mediastinoscopy with biopsy.

At our institution, multi-plane sectioning was typically not performed unless requested by the surgeon. The multi-plane sectioning technique as described in this study was modeled after a technique previously reported by Nichols and colleagues [1]. The difference between the standard sectioning and multi-planar techniques lies in the number of levels and hence the depth of tissue analysis per sample. Because a biopsy may include normal conjunctiva in addition to follicles, and granulomas are not uniformly distributed, an increase in the number of sections through different levels at different depths may enhance the probability of detecting granulomas. The number of patients in this series is small, and the absolute number of sarcoid diagnoses picked up by multi-plane sectioning is therefore small. However, if given a larger number of patients, this increased yield may be clinically important. Since there is no increased risk to patients, and little increased effort required by the pathologist, we believe that the information presented in this small series supports the use of multi-plane sectioning.

We observed that two of the three negative biopsy patients had been on topical corticosteroid therapy prior to biopsy. Corticosteroid therapy has previously been reported to reduce the incidence of conjunctival granulomas [18,21]. While this series was too small to find a statistically significant effect of topical corticosteroid on biopsy result, it may be prudent to discontinue topical corticosteroid prior to conjunctival biopsy to optimize biopsy yield, although the ideal length of time of discontinuation is yet to be determined.

Patients in this series were more likely to have positive biopsy results if they had chest imaging consistent with sarcoidosis. In addition, 75% of those with positive biopsies and available lab results had elevated serum markers consistent with granulomatous inflammation (ACE and/or lysozyme).

While biopsy of pulmonary lesions could have resulted in tissue diagnosis of sarcoidosis, conjunctival biopsy carries a lower risk of morbidity and, if possible, should be considered before more invasive procedures.

## Conclusions

Directed conjunctival biopsy is a simple, relatively non-invasive procedure that allows for tissue diagnosis of sarcoidosis. Although, limitations of our paper include the small number of patients and the use of two ocular pathologists; our data suggest that a positive directed biopsy yield is higher in patients not on topical corticosteroids, and that multi-plane sectioning may increase biopsy yield when standard sectioning techniques are negative.

## Abbreviations

CXR: chest X-ray; CT: computed tomography.

## Competing interests

The authors declare that they have no competing interests.

## Authors’ contributions

KMB is a medical retina fellow at the USC-Doheny Eye Institute. JMG is a vitreoretinal fellow at the University of Chicago. They were involved in the acquisition and interpretation of data and drafted the manuscript. SSP is an assistant professor of ophthalmology and director of uveitis service at the Weill Cornell Medical College. He was involved in the data analysis and revision of the manuscript. AYL is an assistant professor of ophthalmology and pathology and the director of the ophthalmic pathology laboratory at the University of Illinois at Chicago. DPE is a professor of ophthalmology and pathology at Wilmer Eye Institute and the director of research at King Khaled Eye Specialist Hospital in Saudi Arabia. AYL and DPE were involved in the interpretation of data and revision of the manuscript. DAG is a professor of ophthalmology and director of the uveitis service at Northwestern University Feinberg School of Medicine. She was involved in the conception and design of the study and revision of the manuscript. All authors have read and given final approval of the version of manuscript to be published and agree to be accountable for all aspects of the work.
